# Laser isotope separation of ^176^Lu through off-the-shelf lasers

**DOI:** 10.1038/s41598-021-97773-8

**Published:** 2021-09-14

**Authors:** M. V. Suryanarayana, M. Sankari

**Affiliations:** Bhabha Atomic Research Centre, Visakhapatnam, Andhra Pradesh 530 011 India

**Keywords:** Atomic and molecular interactions with photons, Electronic structure of atoms and molecules, Quantum optics, Theoretical physics, Atomic and molecular physics, Optical physics

## Abstract

We propose a novel and simple method for the laser isotope separation of ^176^Lu a precursor for the production of ^177^Lu medical isotope. The physics of the laser-atom interaction has been studied through the dynamics of the atomic level populations using the density matrix formalism. It has been shown that a combination of cw excitation lasers and pulsed ionization laser can be used for the laser isotope separation of ^176^Lu. The optimum conditions for the efficient and selective separation of ^176^Lu have been derived by studying the time evolution of level population under laser excitation. It has also been shown that, it might be possible to produce ~ 100% enriched ^176^Lu isotope at a rate of 5 mg/h, which is higher than all previously reported methods so far. The isotope separation process proposed can be easily adopted using off-the-shelf lasers, for similar atomic systems.

## Introduction

Atomic Vapor Laser Isotope Separation (AVLIS) of actinides (particularly Uranium) has applications in nuclear industry and defence^1^; therefore, a lot of research has been carried out to achieve the above objective. The technology thus developed is largely limited to a handful of technologically advanced nations and strongly controlled due to proliferation risks. Most of the AVLIS activity is centred on utilization of high repetition rate Copper Vapor Laser (CVL) or Solid-State Laser (SSL) pumped dye laser systems for the excitation and ionization of the desired isotope(s). The high pulse repetition frequency (PRF) (10 kHz or higher) of these lasers ensures high interaction efficiency with the atomic vapor enabling the production of the desired isotopes in large scales. It has been realised that AVLIS technology has the ability to play a pivotal role in nuclear medicine^2^.

^177^Lu isotope has gained importance in the targeted radionuclide therapy of small tumors and metastatic lesions^3,4^. ^177^Lu can be produced in a nuclear reactor by irradiation of its precursor (or parent) ^176^Lu isotope. The cross-section ^4^ of this nuclear reaction is 2090 b. ^177^Lu having a half-life of 6.65 d decays to ^177^Hf emitting β-particles with energies 497 keV (76%), 384 keV (9.7%) and 176 keV (12%). The ^177^Hf which is formed in nuclear excited states decays to the ground state emitting low energy γ-radiation with energies 208 keV (11%) and 113 keV (6.6%). With mean penetration depths of β-particles in sub-mm, the radionuclide ^177^Lu is capable of delivering energy in small volumes, thus having localized cytotoxic effect on the disease cells. The γ-radiation is useful for imaging and studies of bio-distribution and excretion kinetics of the infused radionuclide. Due to these advantages, ^177^Lu has evolved as the most effective theranostic (therapeutic + diagnostic) isotope in nuclear medicine. However, natural Lu cannot be used for the production of ^177^Lu due to the low natural abundance of its precursor isotope ^176^Lu (2.59%). It is required to be enriched to ≥ 50% level^5^. The typical administered quantity^4^ of ^177^Lu per course for a patient is ~ 7.4 GBq (1.8 µg). Therefore, after taking into account the conversion efficiency, 1 mg of enriched ^176^Lu can be used for the treatment of > 500 patients after irradiation. Despite the requirements in small quantities, currently only a very few laboratories in the world have the ability to produce enriched ^176^Lu isotope leading to the worldwide shortage of this isotope. This is primarily due to the non-availability of the suitable commercial tunable narrow band high repetition rate lasers.

AVLIS exploits the small resonance frequency shifts between the constituent isotopes of an element (called isotope shifts). The lasers in the step-wise ionization are resonantly tuned to the desired isotope causing preferential ionization enabling the separation from the rest of the isotopes. However, laser isotope separation of Lutetium is extremely complex. The nuclear volume changes^6^ in adjacent stable Lu isotopes are small (0.041 fm^2^) resulting in small field shift. Since the mass shift is also small for the heavy element like Lu, the resultant isotope shifts are small. Fortunately, in case of Lutetium, both stable isotopes of Lu (^175^Lu and ^176^Lu) have widely varying nuclear spin, magnetic moment and quadrupole moment values. Consequently, ^176^Lu has a widely spread hyperfine spectrum than the interfering ^175^Lu isotope, which can be exploited for the enrichment of ^176^Lu.

Kurchatov Institute^7^, Moscow has reported successful enrichment of ^176^Lu to > 68% with production rate of ~ 4 mg/h. They have used the following photoionization scheme for the enrichment of ^176^Lu.

$$5d6{s}^{2}\,\,^{2}{D}_{3/2} (0.00 {\mathrm{cm}}^{-1}) \stackrel{540.4068 \mathrm{nm}}{\longrightarrow}\,5d6s6p\,\, ^{4}{F}_{5/2}^{o}\left(18504.58 {\mathrm{cm}}^{-1}\right)\stackrel{535.0626 nm}{\longrightarrow }$$

$$5d6s7s \,\,^{4}{D}_{3/2}\left({37,193.98} {\mathrm{cm}}^{-1}\right)\stackrel{618.0061\,\mathrm{nm}}{\longrightarrow}\,{53,375} {\mathrm{cm}}^{-1}Autoionization State \stackrel{}{\longrightarrow }{Lu}^{+}$$

We have recently studied^8^ the isotope selective photoionization of ^176^Lu and optimization of the process conditions for the enrichment of ^176^Lu through the above photoionization scheme using density matrix formalism. The obtained results were in good agreement with the reported experimental data^7^. Further, we have recently reported a new possible photoionization pathway for the enrichment of ^176^Lu using broad band dye lasers^9^. However, most of these schemes are accessible by high repetition rate (10–30 kHz) CVL or SSL pumped dye laser systems. Since these laser systems have been categorised as potential dual use technology equipment, hence they have export restrictions to several countries. Therefore, isotope separation using the above scheme(s) cannot be easily adopted by all the producers of nuclear medicine. This is severely restricting the availability of enriched ^176^Lu isotope resulting in global shortage of the ^177^Lu medical isotope.

In order to enhance the production of enriched ^176^Lu isotope, it is required to look into the photoionization schemes which are easily accessible by off-the-shelf lasers. In the present work, we have theoretically studied the possibility of using off-the-shelf lasers for the enrichment of^176^Lu isotope.

### Photoionization of Lu

Lutetium has two stable isotopes. They are ^175^Lu (97.41%) and ^176^Lu (2.59%). It has a ground state configuration of 5d6s^2^ ^2^D_3/2_ (0.00 cm^−1^) and has an ionization potential of 43,762.60 cm^−1^. From the known energy levels^10^ of Lu, it is possible to formulate innumerable multi-step photoionization schemes. Apart from the well-known first excitation transitions 540.4068 nm and 573.8130 nm accessible by dye lasers, several strong first excitation transitions^11^ exist between the wavelength range 272–495 nm. Among them, the transitions between the wavelength range of 350–495 nm are accessible by continuous wave (cw) diode laser or second harmonic of the Ti:Sapphire lasers. The hyperfine structure^13^ of Lu isotopes of several photoionization schemes originating from these transitions have been studied for the relative separation of the hyperfine components of the constituent isotopes. A list of possible photoionization schemes which showed good separation of the most intense hyperfine component of ^176^Lu from the hyperfine structure of ^175^Lu have been tabulated in Table 1. The requisite wavelengths for these schemes are accessible by commercially available diode lasers or Ti:Sapphire lasers (fundamental frequency or through second harmonic generation). Among them, the following excitation transition has been considered for further discussion.

**Table 1:** Table of two step photoionization schemes for the laser isotope separation of Lu isotopes. Energy level data obtained from Ref. ^10^.

Ground state	Vacuum wavelength of the first excitation laser (nm)	First excited state	Vacuum wavelength of the second excitation laser (nm)	Second excited state
5d6s2 ^2^D_3/2_ (0.00 cm^−1^)	471.8012	5s6s6p ^4^D^0^_3/2_ (21,195.37 cm^−1^)	710.27	6s6p^2 4^P_5/2_ (35,274.5 cm^−1^)
			1143.83	5d^2^6s ^2^P_3/2_ (29,937.9 cm^−1^)
	465.9316	5d6s6p ^2^D^0^_5/2_ (21,462.38 cm^−1^)	1179.87	5d^2^6s ^2^P_3/2_ (29,937.9 cm^−1^)
	451.9823	5d6s6p ^2^D^0^_3/2_ (22,124.76 cm^−1^)	760.47	6s6p^2 4^P_5/2_ (35,274.5 cm^−1^)
			854.21	6s6p^2 4^P_3/2_ (33,831.46 cm^−1^)
	411.3857	5d6s6p ^4^P^0^_3/2_ (24,308.09 cm^−1^)	911.87	6s6p^2 4^P_5/2_ (35,274.5 cm^−1^)

### 465 nm–1179 nm scheme

$$5d6{s}^{2}\,^{2}{D}_\frac{3}{2}\left(0.00 \,\,{\mathrm{cm}}^{-1}\right)\stackrel{465.9316\,\,\mathrm{nm}}{\longrightarrow} 5d6s6p \,\,^{2}{D}_\frac{5}{2}^{o}\left(21462.38 {\mathrm{cm}}^{-1}\right)\stackrel{1179.87 \mathrm{nm}}{\longrightarrow}$$

$${5d}^{2}6s\,^{2}{P}_\frac{3}{2}\left({29,937.9} {\mathrm{cm}}^{-1}\right)\stackrel{}{\longrightarrow}\,\,Autoionization\,\,State\stackrel{}{\longrightarrow}{Lu}^{+}$$

Photoionization of Lu through pulsed blue dye lasers^14^ for the first step excitation transition was reported way back in 1981. Later, photoionization schemes originating from UV-blue wavelength region^15,16^ have been studied for the photoionization of Lu using second and third harmonics of the pulsed Ti:Sapphire lasers to access the requisite wavelengths. However, such lasers have linewidths in several GHz, hence, cannot be easily adopted for the isotope separation of Lu.

Utilization of diode lasers for isotope separation was first reported by Olivares et al.^17^, wherein two-step photoionization method was used for the isotope separation of Li isotopes. Recently Matsuoka et al.^18^ have studied isotope separation of ^48^Ca using diode lasers. In both cases ionization has been carried out by a non-resonance ionization process using pulsed lasers. Further, applicability of such a method for large scale separations has not been stated.

The isotope shifts for the first and second excitation transitions of Lu are typically in the range of 400 MHz and 100 MHz respectively. From the hyperfine structure constant data^12,13^ (Table 2), the frequency positions of the two-step hyperfine excitation pathways have been calculated and tabulated in Table 3. As it can be seen from the Table 3, the most intense hyperfine excitation pathway 17/2–19/2–17/2 at the frequency position (15,150 MHz, -25,160 MHz) of ^176^Lu is well separated from the nearest and the most intense hyperfine excitation pathway 5–6-5 which is at the frequency position (11,100 MHz, −17,720 MHz)of ^175^Lu.

**Table 2:** Table of HFS coupling constants of lutetium energy levels for the 465–1179 nm photoionization scheme.

Energy level	^175^Lu		^176^Lu		References
	A (MHz)	B (MHz)	A (MHz)	B (MHz)	
5d6s^2 2^D_3/2_ (0.00 cm^−1^)	194.331617 (89)	1511.398650 (686)	137.920537 (117)	2132.296936 (2473)	Ref. ^12^
5d6s6p ^2^D^0^_5/2_ (21,462.38 cm^−1^)	46.64 (0.05) mK 1398.2 (1.5)^a^	−23.0 (1) mK −689 (30)^a^	992.3 (1.5)^b^ (computed)	−972 (30)^a^ (computed)	Ref. ^13^
5d^2^ 6 s ^2^P_3/2_ (29,937.9 cm^−1^)	−34.05 (0.1) mK −1021 (3)^a^	−39.7 (0.5) mK −1190 (15)^a^	−725 (3)^b^ (computed)	−1679 (15)^a^ (computed)	

^a^Converted from mK to MHz.

^b^Calculated from the ratios of the A, B constants of the energy levels for each isotope.

**Table 3:** Table of the hyperfine excitation pathways of ^175^Lu and ^176^Lu for the 465–1179 nm photoionization pathway (the frequency position is with reference to the centre of gravity of ^176^Lu isotope).

S No	^175^Lu						^176^Lu					
	F1–F2–F3	Frequency Position of the first hyperfine transition (MHz)	Normalised intensity	Frequency Position of the second hyperfine transition (MHz)	Normalised intensity	Sum two photon frequency position (MHz)	F1–F2–F3	Frequency Position of the first hyperfine transition (MHz)	Normalised intensity	Frequency Position of the second hyperfine transition (MHz)	Normalised intensity	Sum two photon frequency position (MHz)
1	2–1–2	−15,160	23.1	22,350	23.1	7190	11/2–9/2–11/2	−19,360	50.0	28,290	50.0	8930
2	2–2–2	−12,170	23.1	19,360	23.1	7190	11/2–11/2–11/2	−13,600	29.5	22,530	29.5	8930
3	2–2–3	−12,170	23.1	17,150	15.4	4980	11/2–11/2–13/2	−13,600	29.5	18,840	30.5	5240
4	3–2–2	−11,670	15.4	19,360	23.1	7690	13/2–11/2–11/2	−13,200	30.5	22,530	29.5	9330
5	3–2–3	−11,670	15.4	17,150	15.4	5480	13/2–11/2–13/2	−13,200	30.5	18,840	30.5	5640
6	2–3–2	−7750	11.5	14,940	11.5	7190	15/2–13/2–11/2	−7290	15.6	15,850	10.5	8560
7	2–3–3	−7750	11.5	12,730	33.7	4980	15/2–13/2–13/2	−7290	15.6	12,150	43.9	4860
8	2–3–4	−7750	11.5	8990	8.7	1240	15/2–13/2–15/2	−7290	15.6	6930	15.6	−360
9	4–3–2	−7600	8.7	14,940	11.5	7340	11/2–13/2–11/2	−6920	10.5	15,850	10.5	8930
10	4–3–3	−7600	8.7	12,730	33.7	5130	11/2–13/2–13/2	−6920	10.5	12,150	43.9	5230
11	4–3–4	−7600	8.7	8990	8.7	1390	11/2–13/2–15/2	−6920	10.5	6930	15.6	10
12	3–3–2	−7260	33.7	14,940	11.5	7680	13/2–13/2–11/2	−6520	43.9	15,850	10.5	9330
13	3–3–3	−7260	33.7	12,730	33.7	5470	13/2–13/2–13/2	−6520	43.9	12,150	43.9	5630
14	3–3–4	−7260	33.7	8990	8.7	1730	13/2–13/2–15/2	−6520	43.9	6930	15.6	410
15	5–4–3	−3900	3.2	6980	31.7	3080	17/2–15/2–13/2	−2220	5.4	4610	30.6	2390
16	5–4–4	−3900	3.2	3240	34.3	−660	17/2–15/2–15/2	−2220	5.4	−620	44.0	−2840
17	5–4–5	−3900	3.2	−2720	3.2	−6620	17/2–15/2–17/2	−2220	5.4	−7790	5.4	−10,010
18	4–4–3	−1850	34.3	6980	31.7	5130	15/2–15/2–13/2	250	44.0	4610	30.6	4860
19	4–4–4	−1850	34.3	3240	34.3	1390	15/2–15/2–15/2	250	44.0	−620	44.0	−370
20	4–4–5	−1850	34.3	−2720	3.2	−4570	15/2–15/2–17/2	250	44.0	−7790	5.4	−7540
21	3–4–3	−1510	31.7	6980	31.7	5470	13/2–15/2–13/2	1020	30.6	4610	30.6	5630
22	3–4–4	−1510	31.7	3240	34.3	1730	13/2–15/2–15/2	1020	30.6	−620	44.0	400
23	3–4–5	−1510	31.7	−2720	3.2	−4230	13/2–15/2–17/2	1020	30.6	−7790	5.4	−6770
24	5–5–4	3060	23.7	−3730	60.9	−670	17/2–17/2–15/2	6110	29.6	−8940	60.4	−2830
25	5–5–5	3060	23.7	−9680	23.7	−6620	17/2–17/2–17/2	6110	29.6	−16,120	29.6	−10,010
26	4–5–4	5120	60.9	−3730	60.9	1390	15/2–17/2–15/2	8580	60.4	−8940	60.4	−360
27	4–5–5	5120	60.9	−9680	23.7	−4560	15/2–17/2–17/2	8580	60.4	−16,120	29.6	−7540
28	5–6–5	11,100	100.0	−17,720	100.0	−6620	17/2–19/2–17/2	15,150	100.0	−25,160	100.0	−10,010

**Figure 1:**
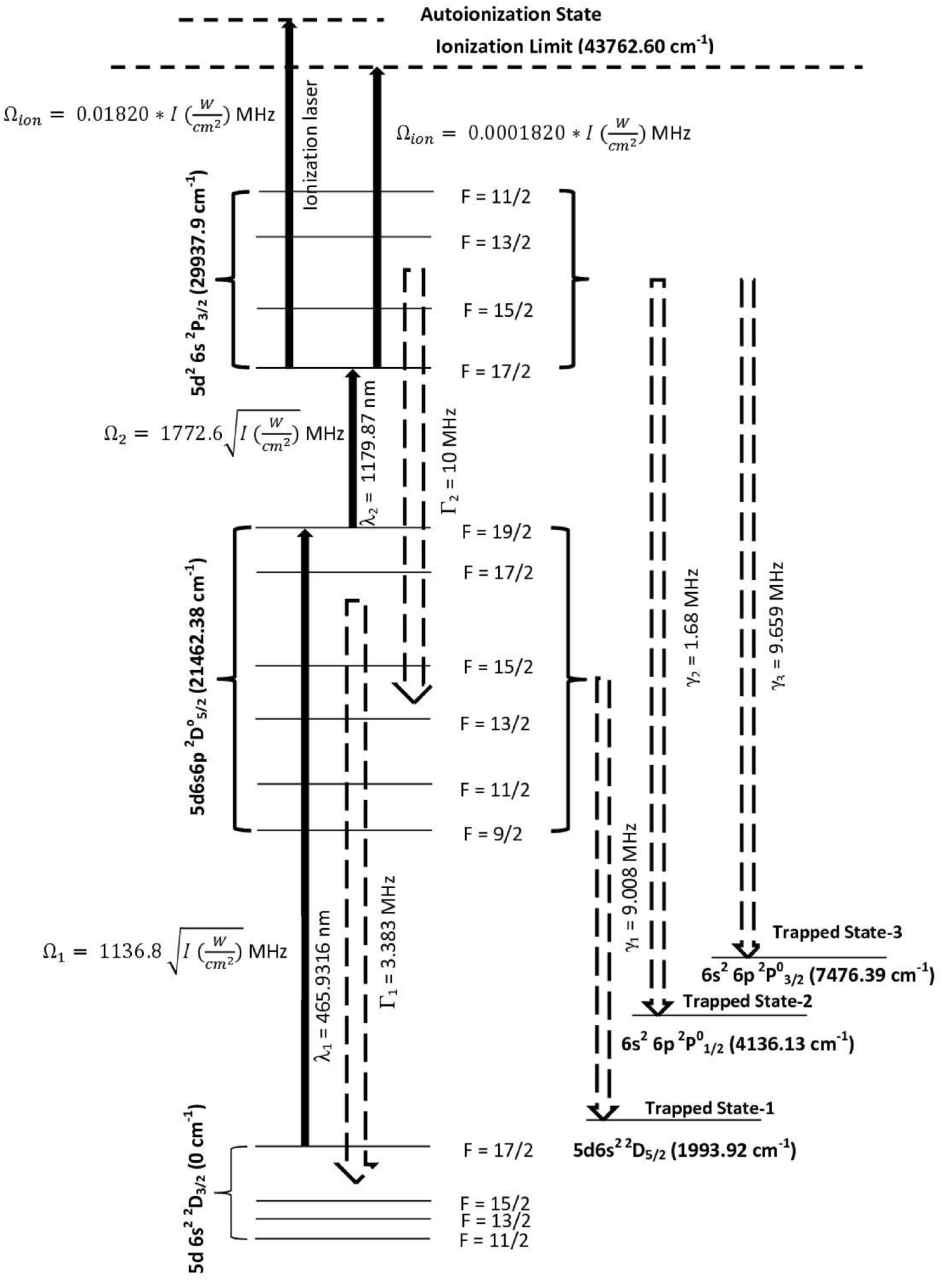
Schematic of the photionization pathway of ^176^Lu (not to scale). The excitation rate (Rabi frequency), decay rate to lower level and decay rate to trapped levels are represented by Ω, Γ and γ respectively^11^. The radiative decay rate for the 1179.87 nm is taken as 10 MHz. Please see main section for the discussion.

The schematic of the photoionization scheme is shown in Fig. 1. The atoms in the ground state 5d6s^2 2^D_3/2_ (0.00 cm^−1^) are excited to the 5d6s6p ^2^D^0^_5/2_ (21,462.38 cm^−1^) level using the 465.9316 nm laser and atoms from this level are further excited to the 5d^2^6s ^2^P_3/2_ (29,937.9 cm^−1^) level using the 1179.87 nm laser, which are ionized using an ionization laser through either auto-ionization level or non-resonant ionization. The ionization process is considered as an incoherent process. During the excitation process, due to the finite life-time of the resonant states, the atoms decay either back to their original states or into the trapped states and lost from ionization process. The excitation rates, decay^11^ rates of atoms and the ionization rates are also given in Fig. 1.

The radiative decay rate of the transition 1179.87 nm has not been reported so far. Several transitions originating from the lower levels ranging between 20,000 cm^1^ to 25,000 cm^−1^ have shown^11^ decay rates of about 1 × 10^7^ Hz, therefore; the decay rate is taken as 1 × 10^7^ Hz for the 1179.87 nm transition.

Several articles have been published on the even parity autoionization states^19–23^ wherein two step laser excitation method has been used. However, very little work has been on the odd parity autoionization states. D’yachkov^7^ et al. have found several odd-parity autoionization states between the energy range 53,067 cm^−1^–53,861 cm^−1^ among which, autoionization transition to 53,375 cm^−1^ was the strongest. Another work^24^ recently reported nineteen new odd parity autoionization levels in the energy range 50,650–51,650 cm^−1^. Using Ti:Sapphire laser for the third excitation step, it is possible to access Rydberg or Auto-ionization state in the range between 39,938 cm^−1^ and 49,560 cm^−1^ from the second excitation level 29,937.9 cm^−1^. Since this is a large energy range, it is very likely to find a suitable autoionization state, nonetheless, can be confirmed only by experiments.

In a laser isotope separation process, the atomic parameters such as isotope shifts, hyperfine structures of the constituent isotopes, radiative lifetimes of levels, branching ratios and ionization cross-section; laser parameters such as power, spectral bandwidth, frequency, pulse width (or interaction time) and delay between the pulses; effusive atom source parameters such as atomic velocity distribution and angular divergence (collimation ratio), all have a complex interplay on the ionization efficiency and selectivity of the photoionization process.

The coherent laser–atom interactions can be accurately described by the density matrix theory^25^. The density matrix equations relevant for the pulsed two-step excitation process have been published in our recent article^8^. When, the atoms traverse perpendicular to the spatially overlapped co-propagating laser beams, for all CW lasers case, the laser-atom interaction time is determined by the atomic velocity and the laser beam diameter. While in case of CW laser excitation followed by pulsed laser ionization, the laser-atom interaction time is determined by the temporal width of the pulsed laser. The coupled density matrix elements have been integrated for the entire laser-atom interaction time determined as described above. The effect of linewidth of excitation lasers has been incorporated according to the phase diffusion model as discussed in our recent article^8^.

In an AVLIS process, when the first and second excitation lasers are tuned to the frequencies ν_1_ and ν_2_ respectively, isotope selectivity is defined as

1 $${\text{Isotope Selectivity S }}({\upnu }_{1},{\upnu }_{2})=\left(\frac{{\upeta }_{{176}_{\mathrm{Lu}}}}{{\upeta }_{{175}_{\mathrm{Lu}}}}\right)$$

where, η is the ionization efficiency of the isotope.

When the abundance of the isotopes is not equal, one needs to normalize the selectivity with the abundance of the constituent isotopes; thus, isotope ratio enhancement factor is defined as

2 $${\text{Isotope Ratio Enhancement Factor IRE }}({\upnu }_{1},{\upnu }_{2})=\left(\frac{{\upeta }_{{176}_{\mathrm{Lu}}}}{{\upeta }_{{175}_{\mathrm{Lu}}}}\right)\times \left(\frac{{\mathrm{f}}_{{176}_{\mathrm{Lu}}}}{{\mathrm{f}}_{{175}_{\mathrm{Lu}}}}\right)$$

where, f is the fractional abundance of the isotope.

Degree of enrichment is calculated using the expression

3 $${\text{Degree of enrichment }}(\mathrm{\%}) =\left\{\frac{{\upeta }_{{176}_{\mathrm{Lu}}}{\times \mathrm{f}}_{{176}_{\mathrm{Lu}}}}{\left({\upeta }_{{175}_{\mathrm{Lu}}}{\times \mathrm{f}}_{{175}_{\mathrm{Lu}}}+{\upeta }_{{176}_{\mathrm{Lu}}}{\times \mathrm{f}}_{{176}_{\mathrm{Lu}}}\right)}\right\}\times 100$$

## Results and discussion

For a source temperature of 1600 °C, the most probable velocity of ^176^Lu is 421.8 m/s. At this temperature the Doppler broadening of the 465.9316 nm transition is ~ 1500 MHz. Let us consider that the atom source to have a length-to-diameter (L/D) ratio of 5 corresponding to the full-angle divergence (θ) of 22.6°. For the orthogonal laser-atom interaction, Doppler broadening along the laser propagation axis reduces to ~ 565 MHz. Let us also consider that the laser atom interaction region^26^ to have dimensions of 10 mm diameter and 270 mm long.

First, we look at the possibility of using cw lasers for excitation and ionization of ^176^Lu isotope.

### Excitation and ionization using cw lasers

As mentioned earlier, the primary aim of the present work is to investigate the enrichment of ^176^Lu through off-the-shelf lasers. A detailed survey has been carried out on the availability of laser systems in the wavelength ranges indicated in Table 1. Blue diode laser systems within the wavelength range of the first excitation transition are available with a typical cw power of 30 mW; while diode lasers with accessible wavelengths of the second excitation transition have a typical power of 120 mW. Therefore, we set the limit to the powers of first and second excitation lasers as 30 mW and 120 mW corresponding to the power densities of 38.2 mW/cm^2^ and 153 mW/cm^2^respectively. The saturation intensities of the transitions 465.9316 nm and 1179.87 nm are 13 mW/cm^2^ and 3 mW/cm^2^ respectively which can be easily achieved using the available cw lasers. The frequency of the excitation lasers can be controlled to ~ 1 MHz using the commercially available wavelength meters. Non-resonance ionization can be carried out using the high power 10 W cw lasers which are available in wavelengths of 532 nm (Nd:YAG laser) or 527 nm laser (Nd:YVO_4_ laser). Therefore, we set the limit to the power of ionization laser to 10 W and the corresponding power density is 12.73 W/cm^2^.

**Figure 2:**
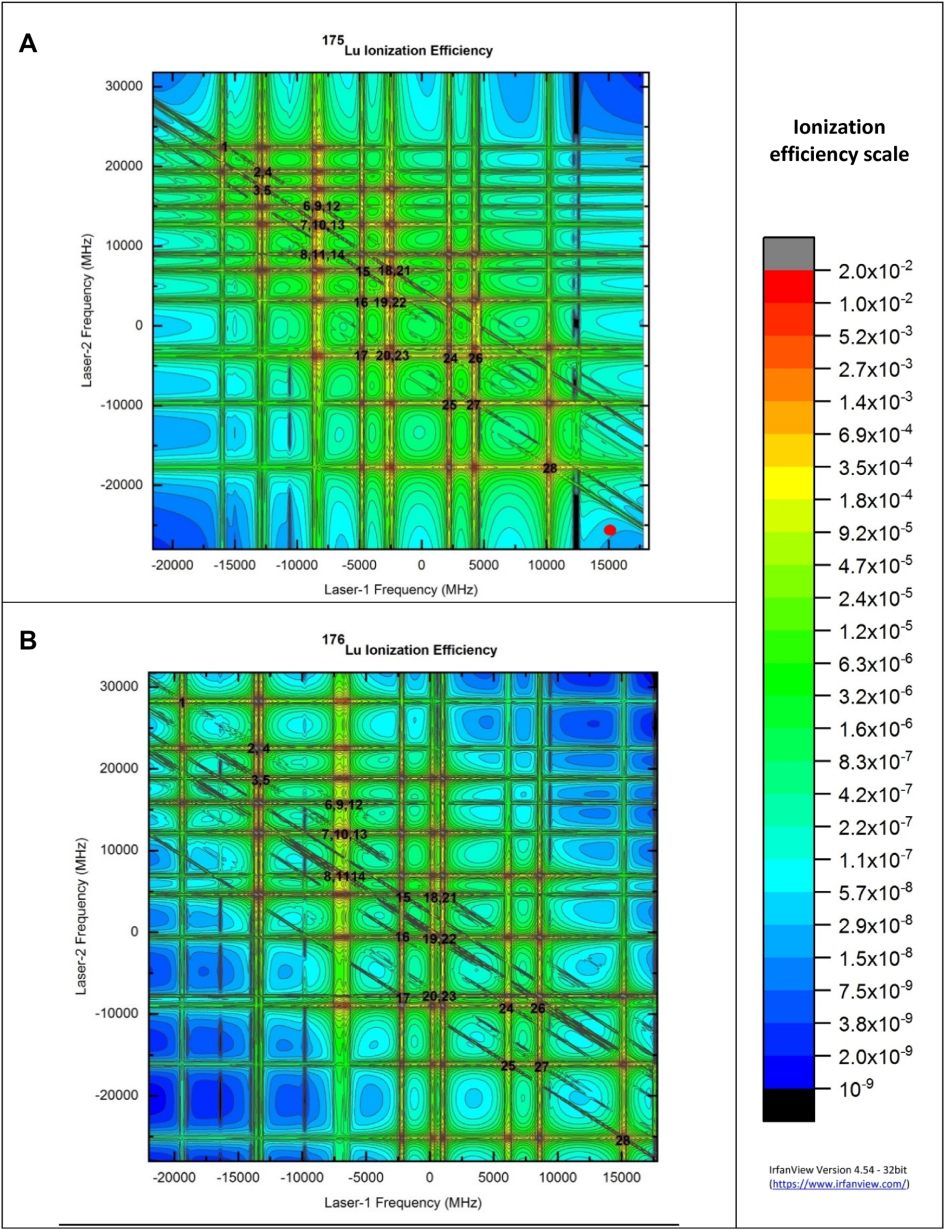
Two dimensional contour plots of ionization efficiencies of **(A)** ^175^Lu and **(B)** ^176^Lu isotopes under Doppler free conditions. The excitation and ionization laser power densities are 0.038 W/cm^2^, 0.153 W/cm^2^ and 12.73 W/cm^2^ respectively. Full angle divergence of the atomic beam is 22.6° and both the excitation lasers are counter propagating having a frequency jitter of 1 MHz. Resonance frequency positions of the hyperfine excitation pathways have been numbered as per Table 3. Resonance frequency position of 17/2–19/2–17/2 (15,150 MHz, -25,160 MHz) of ^176^Lu isotope is shown as red circle in (A).

Calculation of ionization efficiencies of Lu isotopes has been carried out varying the frequencies of the first and second excitation lasers around the resonance of Lu isotopes and the results are plotted as two-dimensional contour plots in Fig. 2. The contours showed resonances in agreement with expected positions (Table 3). Diagonal ridges corresponding to the coherent two photoionization have been observed even at longer detunings due to the strong first and second excitation transitions. The resonance frequency position of the most intense hyperfine excitation pathway 17/2–19/2–17/2 (15,150 MHz, -25,160 MHz) of ^176^Lu lies far away from the most intense hyperfine excitation pathway 5–6–5 (11,100 MHz, −17,720 MHz) of ^175^Lu.

**Figure 3:**
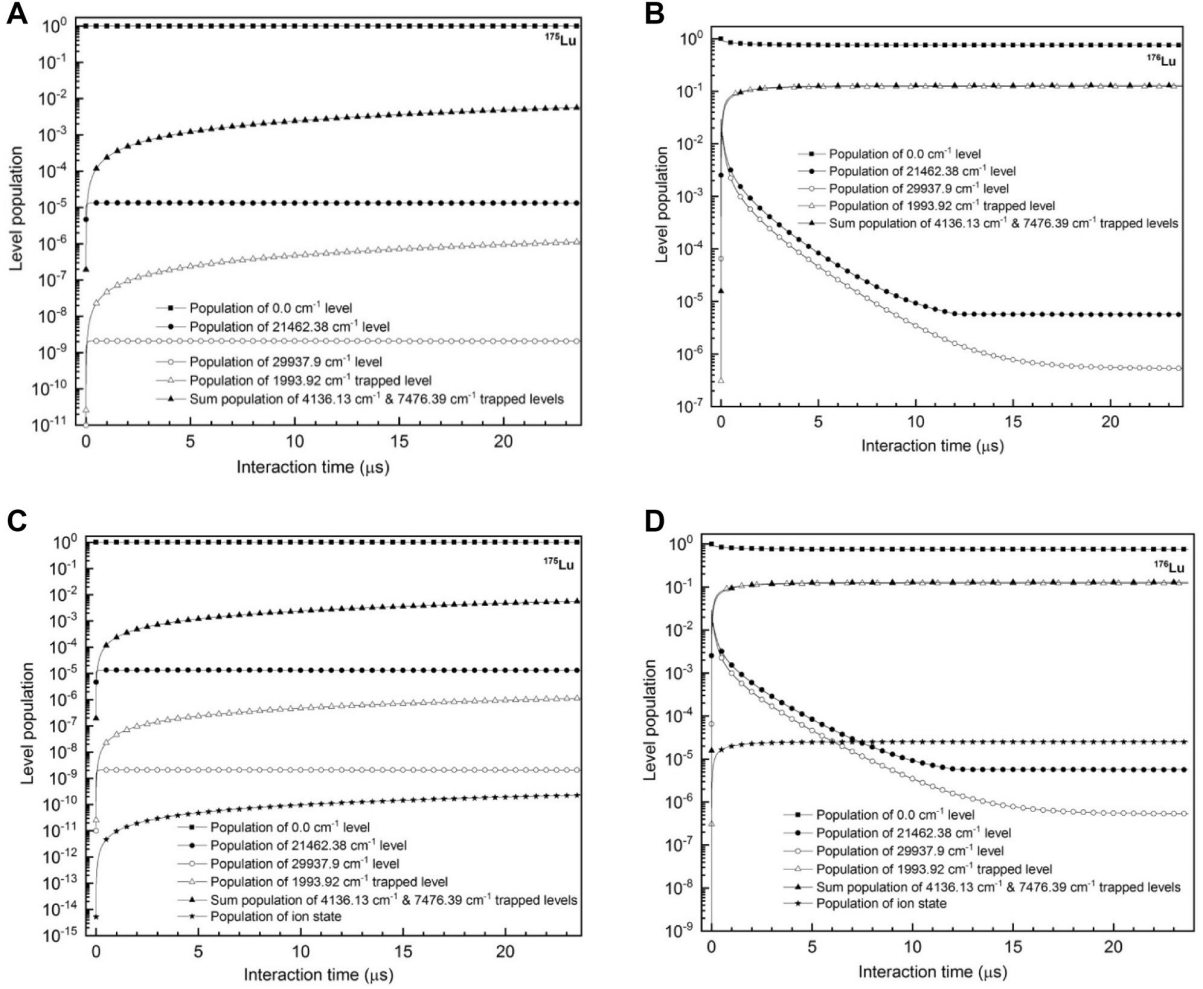
Time evolution of level populations of the ground, excited, ion and trapped states of ^175^Lu **(A,C)** and ^176^Lu **(B,D)** under cw laser excitation. For graphs **(A)** and **(B)** excitation and ionization laser power densities are 0.038 W/cm^2^, 0.153 W/cm^2^ and 0.0 W/cm^2^ respectively. For graphs **(C,D)** excitation and ionization laser power densities are 0.038 W/cm^2^, 0.153 W/cm^2^ and 12.73 W/cm^2^ respectively. Full angle divergence of the atomic beam is 22.6° and both the excitation lasers are counter propagating having frequency jitter of 1 MHz. Excitation lasers are set to the frequencies corresponding to the hyperfine excitation pathway 17/2–19/2–17/2 (15,150 MHz, -25,160 MHz) of ^176^Lu isotope.

Since the most probable velocity of Lu atoms at 1600 °C is 421.8 m/s, for the cw laser beam diameter of 1 cm, the interaction time of atoms with spatially overlapped laser beams is 23.7 µs. The cw lasers typically operate in the fundamental mode TEM_00_ having a Gaussian intensity distribution across the diameter; nevertheless it is possible to convert them into a flat-top intensity distribution using beam shaping optics. For the present work, we have considered lasers to have flat-top intensity distribution across its diameter. First, evolution of level populations with interaction time (Fig. 3A,B) have been calculated setting the excitation lasers to the frequencies corresponding to the hyperfine excitation pathway of 17/2–19/2–17/2 (15,150 MHz, -25,160 MHz) of ^176^Lu and setting the power of the ionization laser to zero. As expected in the case of non-resonant ^175^Lu isotope (Fig. 3A), a very small fraction of atoms which are excited into the upper levels decay into the trapped states. At the end of the interaction, about 0.56% atoms can be found in the 1993.92 cm^−1^ trapped level and rest of the atoms in the ground state 0.00 cm^−1^. On the other hand, for the case of resonant ^176^Lu isotope (Fig. 3B), the atoms from the ground F = 17/2 hyperfine level are excited through the 17/2–19/2–17/2 hyperfine excitation pathway. Since the ionization laser power is set to zero, the atoms excited into the upper hyperfine levels decay to the 1993.92 cm^−1^, 4136.13 cm^−1^ and 7476.39 cm^−1^ trapped levels. Due to the repetitive excitation and decay processes, at the end of the interaction, the entire population of F = 17/2 (30%) is transferred to the trapped levels mentioned above. It is therefore expected that the introduction of cw ionization laser transfers atoms from the excited levels into the ionization continuum. The dynamics of the photoionization process with the introduction of a cw ionization laser is discussed below.

When a cw ionization laser with a power density of 12.73 W/cm^2^ along with the cw excitation lasers interacts with the atomic beam; the ionization efficiency of the non-resonant ^175^Lu isotope (Fig. 3C) reaches to the value of 2.3 × 10^–10^ and the ionization efficiency of resonant^176^Lu isotope (Fig. 3D) reaches to 2.5 × 10^–5^ implying that most of the population excited into the higher levels decays to the trapped states. The low ionization efficiency of the resonant ^176^Lu isotope can be attributed to multiple factors such as low power density of the cw ionization laser and low ionization cross-section (1 × 10^–16^ cm^2^) of the non-resonant ionization process. From the Eqs. (1)–(3), one can calculate the degree of enrichment, which is ~ 100%. Considering the Lu number density of 1 × 10^13^ atoms/cm^3^ and the dimensions^7,26^ of the laser-atom interaction region of 10 mm diameter and 270 mm length, the production rate of enriched ^176^Lu is calculated to be 4.9 µg/h. In comparison to the value of 3.7 mg/h reported by D’yachkov et al.^7^, the production rate in the present case is three orders lower. Therefore, enrichment of ^176^Lu through “*all* cw” laser process is very inefficient. Hence, we have adopted a combination of cw lasers for the excitation process and pulsed laser for the ionization process which is discussed in the next section.

### cw laser excitation and pulsed laser ionization

As discussed in the previous section, the low ionization efficiency of ^176^Lu under cw laser excitation and ionization is due to the low non-resonant ionization cross-section and low peak power of the cw ionization laser. This can be overcome by employing the tunable high repetition rate pulsed laser for the ionization. Theoretical modelling  of cw laser excitation followed by pulsed laser ionization for the isotope selective photoionization of Sr has been studied previously^27^. Currently high repetition rate (up to 10 kHz) Ti:Sapphire lasers are available with the average power of 1 W. The pulse duration of these lasers is 50 ns. These lasers are also having a wavelength tunability between 700 and 1000 nm. The lasers can produce peak power density of 2500 W/cm^2^. Wavelength tunability of these lasers allows atoms to be excited either into high lying Rydberg states followed by electric field ionization or directly into the autoionization levels. Cross-section^28^ for such a process can be in the range of 1 × 10^–14^ cm^2^ which is two-orders higher than the cross-section for the non-resonant ionization process. As a result of high peak power density and the high resonance ionization cross-section, ionization efficiency is expected to be enhanced significantly.

If one carefully observes the time evolution of level populations of the first (21,462.38 cm^−1^) and the second (29,937.9 cm^−1^) excited states of ^176^Lu under cw laser excitation (Fig. 3A,B); one can find that the atomic population from these states starts decaying into the trapped states after ~ 20 ns of its interaction with the excitation lasers. Therefore, for efficient ionization, it is important for the pulsed ionization laser to interact with the *“nascent”* atomic beam within 50 ns of its interaction with the cw lasers. Experimentally, this can be achieved by passing the diode lasers through mechanical choppers and synchronising the pulsed ionization laser with the sync signal of the mechanical chopper (Supplementary material Fig. S1). Alternatively, it is also possible to use high-frequency intensity modulation of narrow-linewidth laser light using fast-switching electro-optical modulators^29^.

**Figure 4:**
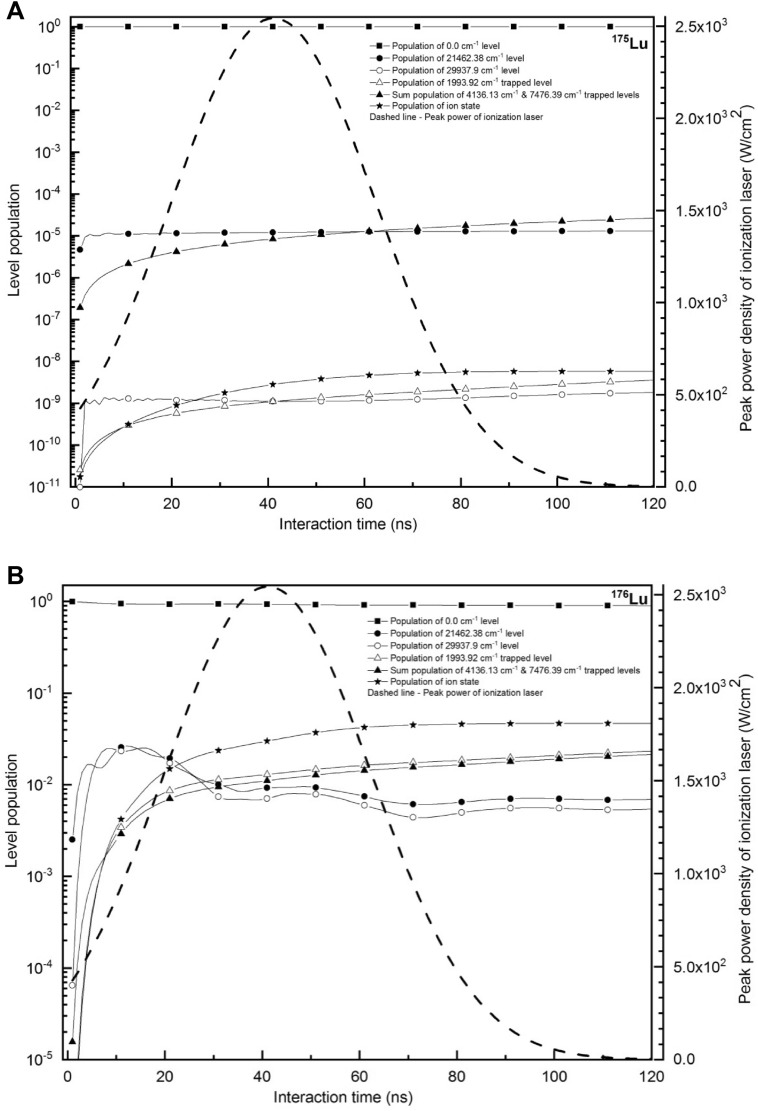
Time evolution of level populations of the ground, excited, ion and trapped states of **(A)** ^175^Lu and **(B)** ^176^Lu under cw laser excitation and pulsed laser ionization. Excitation laser power densities are 0.038 W/cm^2^, 0.153 W/cm^2^ respectively and frequency jitter of excitation lasers is 1 MHz. Peak power density of the ionization laser is 2500 W/cm^2^witha pulsed width of 50 ns. Full angle divergence of the atomic beam is 22.6° and both the excitation lasers are counter propagating. Excitation lasers are set to the frequencies corresponding to the hyperfine excitation pathway 17/2–19/2–17/2 (15,150 MHz, -25,160 MHz) of ^176^Lu isotope.

Evolution of level populations have been calculated for the Lu isotopes by setting the pulsed ionization laser peak power density to 2500 W/cm^2^ with a pulse width of 50 ns. The results are plotted in Fig. 4. It can be seen from the Fig. 4B that, in this case the ionization efficiency of ^176^Lu isotope reaches a value of 4.7 × 10^–2^, as compared to the ^175^Lu ionization efficiency of 5.7 × 10^–9^ (Fig. 4A) corresponding to the degree of enrichment of ~ 100% of the ^176^Lu isotope. Considering the atom number density to be 1 × 10^13^ atoms/cm^3^ and the laser-atom interaction region having dimensions of 10 mm diameter and 270 mm length, the production rate of enriched ^176^Lu is calculated to be 2.7 mg/h. This is comparable to the value of 3.7 mg/h reported by D’yachokov et al.^7^. However, in our case the degree of enrichment is significantly higher (100%) than that has been previously reported (68.4%) value.

In the *“nascent”* atomic beam, the population of the ground state 0.00 cm^−1^ is distributed among the four hyperfine components F = 11/2 to F = 17/2 based on the 2F + 1 rule. Therefore, the F = 17/2 hyperfine level possesses 0.30 (30%) of the population of which 5 × 10^–2^ is ionized. This implies ionization efficiency of 17% of the targeted F = 17/2 hyperfine level population. The ionization efficiency can be further increased by increasing the power of the ionization laser; however, it is not practically viable as tunable high repetition rate lasers with average power > 1 W are not commercially available. Another reason for the low ionization efficiency is due to the large Doppler broadening of the atomic ensemble. As mentioned earlier, for self-collimating atomic beam effusing out of the atom source having L/D ratio of 5 (full angle divergence of 22.6°), the Doppler broadening along the laser propagation axis will be 565 MHz for the 465.9316 nm transition. The diode laser which can be considered as the single frequency system during short interaction times (50 ns), causes a *“hole burning”* in the velocity groups of the Doppler broadened atomic ensemble. The group of atoms having velocities nearly perpendicular to the laser propagation axis induce a very small proportion of velocity induced Doppler shifts. When the excitation laser is tuned to the resonance of a transition, this group of atoms of the resonant isotope are excited and ionized. The remaining atoms experiencing the velocity induced Doppler shifts are less likely to get excited and ionized. This causes low ionization efficiency. One of the ways to overcome such a problem is by increasing the linewidth of the excitation lasers; however, this is associated with the loss in selectivity. Therefore, we have adopted an alternative approach. Angular divergence of the atomic beam can be reduced by increasing the L/D ratio of the atom source which causes reduction in the angular divergence and hence velocity induced Doppler shifts. Using this approach, most of the atoms in the atomic beam can be brought into the resonance by adjusting the L/D ratio of the atom source. Calculation of ionization efficiency and degree of enrichment has been carried out varying the L/D ratio of the atom source. It has been found that for a L/D ratio of 10 corresponding to the full angular divergence of 11.5° enhances the ionization efficiency to 8.8 × 10^–2^ while the degree of enrichment remains at ~ 100%. Such source geometry allows production of ^176^Lu at a rate of 5 mg/h.

### Effect of delay of ionization laser

Effect of delay and delay jitter of the ionization laser pulse with reference to the interaction time has been studied (Supplementary material Fig. S2). Ionization efficiency and the degree of enrichment have been calculated varying delay of the ionization laser with reference to the start time of interaction of cw excitation lasers with *“nascent”* atomic beam. It has been observed that a delay of 50 ns results in highest ionization efficiency. Therefore, it is necessary to set the delay of the ionization laser to 50  ± 10 ns to achieve the ionization efficiency within 90% of its value. This requirement can be easily met with the currently available technological advances in the synchronization electronics.

## Conclusion

We have proposed a novel and simple method for the enrichment of ^176^Lu isotope a precursor to ^177^Lu medical isotope. The physics of the laser-atom interaction has been studied through the dynamics of the atomic level populations using density matrix formalism. It has been shown that a combination of cw excitation lasers and pulsed ionization laser can be used for the laser isotope separation of ^176^Lu. The optimum conditions for the efficient and selective separation of ^176^Lu have been derived by studying the time evolution of level populations during laser excitation. It has been shown that, it might be possible to produce ~ 100% enriched ^176^Lu isotope at a rate of 5 mg/h, which is higher than all previously reported methods so far. The enrichment process proposed can be easily adopted using off-the-shelf lasers for similar atomic systems.

## Method

The isotope shift and hyperfine structure data of natural Lu isotopes have been taken from the literature and the frequency positions of all hyperfine pathways have been derived with reference to centre of gravity of ^176^Lu. A density matrix code for three-step photoionization has been developed. Separate codes have been used for *all cw lasers case;* cw laser excitation followed by pulsed laser ionization case. Ionization efficiencies of natural Lu isotopes have been calculated for various conditions. From the ionization efficiencies, degree of enrichment of ^176^Lu has been derived.

## Supplementary Information

Supplementary Figures.

## Data Availability

All the relevant data is provided with this paper. Any additional data relevant to the work in this paper are available upon reasonable request to the corresponding author.

## Code availability

The code developed is not available for other users.
