# Supplementary material for: Laser isotope separation of 176Lu through off-the-shelf lasers
Source: Sci Rep. 2021 Sep 14;11:18292. doi: 10.1038/s41598-021-97773-8 (PMC8440568; doi:10.1038/s41598-021-97773-8)
Supplement: Supplementary file 1 — Supplementary Figures. [file 41598_2021_97773_MOESM1_ESM.docx]

**Supplementary material**


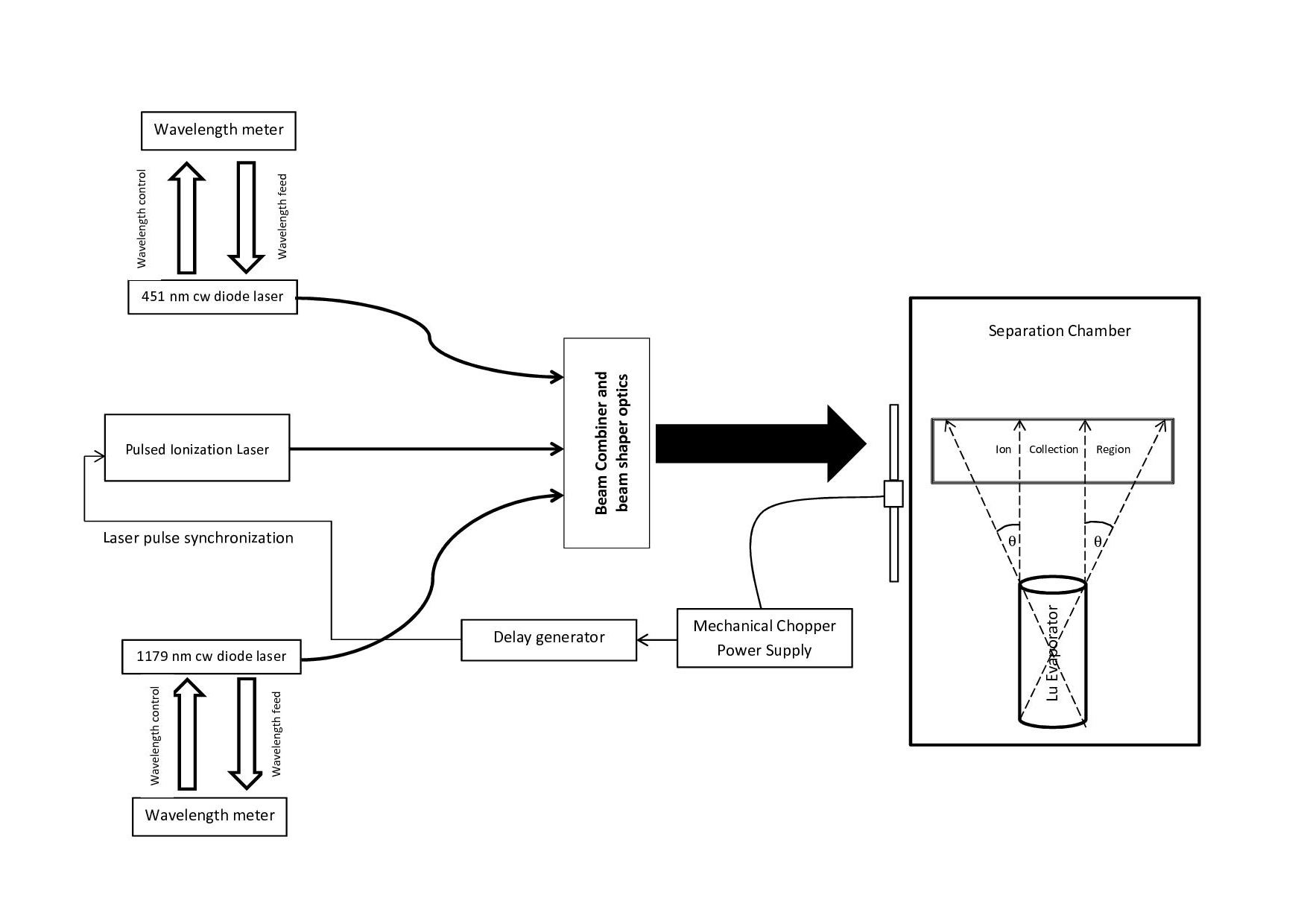


**Figure S1. Schematic diagram of the proposed experimental system for the enrichment of ^176^Lu.**


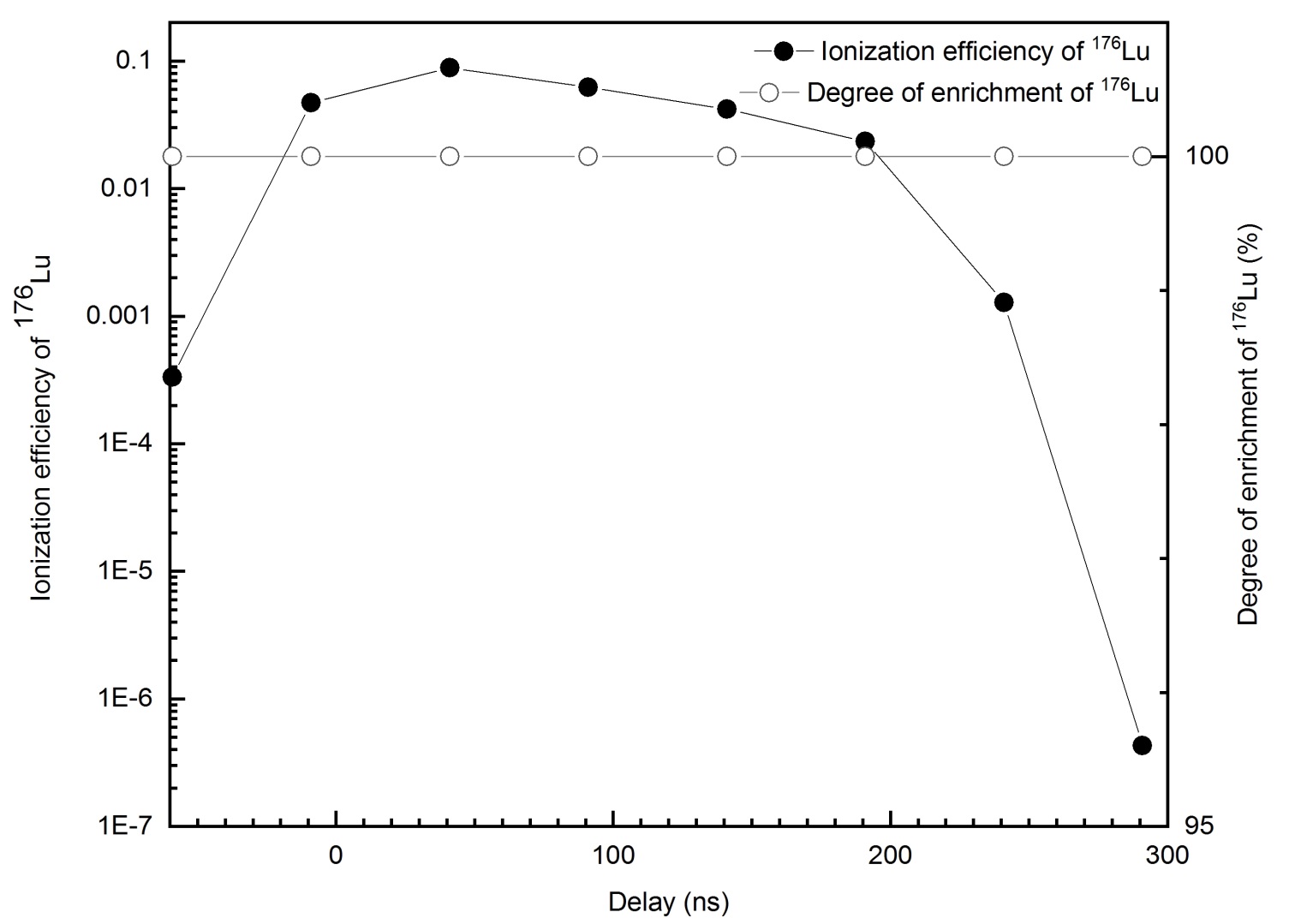


**Figure S2. Effect of delay of the pulsed ionization laser on the ionization efficiency of ^176^Lu isotope and the degree of enrichment.** Excitation laser power densities are 0.038 W/cm^2^, 0.153 W/cm^2^ respectively frequency jitter of excitation lasers is 1 MHz. Peak power density of the ionization laser is 2500 W/cm^2^. Pulse width of the ionization laser is 50 ns. Full angle divergence of the atomic beam is 11.5^0^ and both excitation lasers are counter propagating. Excitation lasers are set to the frequencies corresponding to the hyperfine excitation pathway 17/2-19/2-17/2 (15148.7 MHz, -25159.4 MHz) of ^176^Lu isotope.
